# Study on the Active Constituents and Underlying Mechanisms of Renshen Yangying Decoction Against Angina Pectoris Based on Integrated Serum Pharmacochemistry, Network Pharmacology, Molecular Docking, and Experimental Validation

**DOI:** 10.1155/jamc/6325730

**Published:** 2026-11-03

**Authors:** Ya Huang, Tao Xiong, Haixia Qiu, Rui Cheng, Yule Luo, Xingdong Wu

**Affiliations:** ^1^ Jinsha County Chinese Medicine Hospital Jinsha Guizhou 551800 China; ^2^ School of Pharmacy Zunyi Medical University Zunyi Guizhou 563000 China zmc.edu.cn

**Keywords:** active constituents, angina pectoris, mechanism of action, network pharmacology, Renshen Yangying decoction (RYD), serum components

## Abstract

**Objective:**

This study aims to elucidate the pharmacological basis and underlying molecular mechanisms of Renshen Yangying decoction (RYD) in alleviating angina pectoris.

**Methods:**

UHPLC–Q‐TOF‐MS was used to profile chemical constituents in RYD and RYD‐medicated serum. Compound identification was performed using a custom database, literature, reference standards, and the GNPS platform. Network pharmacology predicted disease‐related targets and pathways, followed by molecular docking and in vitro AC16 cardiomyocyte assays for validation.

**Results:**

A total of 179 constituents in the decoction and 37 absorbed prototype compounds (mainly flavonoids, organic acids, alkaloids, and coumarins) were identified. 282 shared targets with angina were obtained, yielding 10 core targets (e.g., AKT1, TNF, BCL2, STAT3, and EGFR). Four main compounds (naringenin, schisantherin B, schisandrol A, and quercetin) were predicted to modulate EGFR, IGF‐1R, and PDGFR‐β pathways. Naringenin was experimentally confirmed to alleviate pathological damage by suppressing inflammatory responses.

**Conclusion:**

RYD exerts antiangina effects through a multicomponent, multitarget, multipathway mechanism involving EGFR, PI3K–Akt, and PDGFR‐β signaling, with naringenin, schisantherin B, schisandrol A, and quercetin as major active constituents. These findings provide mechanistic insights supporting the clinical application of RYD.

## 1. Introduction

Driven by rapid socioeconomic development and lifestyle transitions, the incidence and mortality rates of coronary heart disease have been rising steadily. Angina pectoris, a prevalent clinical syndrome driven by transient myocardial ischemia and hypoxia secondary to insufficient coronary blood flow, imposes a significant global public health burden [1]. Clinically manifested as paroxysmal and squeezing chest pain, its underlying pathophysiology is primarily attributed to an imbalance between myocardial oxygen supply and demand. Prolonged or recurrent episodes are associated with a markedly elevated risk of severe cardiovascular events, such as myocardial infarction and heart failure, thereby severely compromising patient prognosis [1]. Contemporary therapeutic strategies for angina pectoris predominantly focus on mitigating myocardial ischemia, inhibiting platelet aggregation, and promoting anticoagulation [2]. However, conventional pharmacological interventions are often hampered by challenges like drug intolerance and resistance, whereas surgical revascularization may fail to adequately prevent exertional chest pain [3]. Emerging evidence suggests that natural medicines exhibit remarkable therapeutic efficacy in managing angina pectoris [4–6], serving as promising adjunctive therapies for both primary and secondary prevention.

Renshen Yangying decoction (RYD), a classic traditional Chinese medicine (TCM) formulation originating from the *Taiping Huimin Heji Jufang*, is composed of multiple botanical drugs, including Astragali Radix, Schisandrae Chinensis Fructus, Dendrobii Caulis, Atractylodis Macrocephalae Rhizoma, Chuanxiong Rhizoma, Angelicae Sinensis Radix, Salviae Miltiorrhizae Radix et Rhizoma, Citri Reticulatae Pericarpium, Paeoniae Radix Rubra, Codonopsis Radix, Puerariae Lobatae Radix, Poria, Ophiopogonis Radix, Rehmanniae Radix Praeparata, Scrophulariae Radix, and Polygalae Radix. It possesses well‐documented properties in alleviating myocardial inflammation and enhancing immune activity, making it highly applicable for treating ischemia‐induced myocardial injuries [7]. Currently, RYD is frequently employed in clinical practice to alleviate angina pectoris [8]. Nevertheless, its complex chemical profile and pharmacological mechanisms remain largely elusive. Although the chemical compositions of its individual botanical constituents have been extensively studied, dynamic chemical transformations during the co‐decoction process mean that the holistic profile of the final formulation cannot be accurately deduced by merely summating individual herbal components. Therefore, systematically elucidating its pharmacological basis and underlying molecular mechanisms is of profound scientific significance for advancing the modern clinical application of this classic formula. To address this knowledge gap, this study employed ultra‐performance liquid chromatography coupled with quadrupole time‐of‐flight mass spectrometry (UHPLC–Q‐TOF‐MS) to comprehensively characterize the chemical constituents of RYD and its absorbed compounds in medicated serum [9, 10]. Subsequently, an integrative network pharmacology approach was used to predict the key active constituents and potential therapeutic mechanisms of RYD against angina pectoris [11]. Finally, molecular docking simulations and in vitro AC16 cell experiments were performed to experimentally validate the interactions between core active constituents and therapeutic targets [12, 13]. Ultimately, this study provides a robust scientific rationale for the clinical application of RYD and offers a theoretical reference for the development of novel TCM‐based therapeutics for angina pectoris treatment.

## 2. Materials

### 2.1. Instruments

The primary analytical and experimental instruments utilized in this study included a Shimadzu ultra‐high‐performance liquid chromatography (UHPLC) system (Shimadzu Corporation, Japan); a SCIEX X500R Q‐TOF mass spectrometer (Q‐TOF‐MS) system (AB SCIEX, USA); an ME204E analytical balance (Mettler Toledo, Switzerland); an ultrasonic cleaner (Xiaomei Ultrasonic Instrument Co., Ltd., China); an ultrapure water system (Waterpur, China); an H2050R high‐speed refrigerated centrifuge (Hunan Xiangyi Laboratory Instrument Development Co., Ltd., China); a vortex mixer (Shanghai Titan Technology Co., Ltd., China); a real‐time fluorescence quantitative PCR (RT‐qPCR) system (Bale Technology Co., Ltd., China); a microspectrophotometer (Hangzhou Youmi Instrument Co., Ltd., China); and a thermal cycler (Hangzhou LongGene Scientific Instruments Co., Ltd., China).

### 2.2. Reagents and Drugs

RYD consists of the following botanical materials: *Astragalus membranaceus, Schisandra chinensis, Dendrobium nobile, Atractylodes macrocephala, Ligusticum chuanxiong, Angelica sinensis, Salvia miltiorrhiza, Citrus reticulata, Paeonia lactiflora, Codonopsis pilosula, Pueraria lobata, Poria cocos, Ophiopogon japonicus, Rehmannia glutinosa, Scrophularia ningpoensis,* and *Polygala tenuifolia.* These raw materials were supplied by Jinsha County Hospital of Traditional Chinese Medicine (Guizhou, China) and authenticated by Associate Professor Wang Bo from Guizhou University of Traditional Chinese Medicine. All botanical species conformed to the quality standards and descriptions recorded in the 2025 edition of the *Pharmacopoeia of the People’s Republic of China*.

The human cardiomyocyte cell line AC16 and DMEM/F12 medium were purchased from YOBIBIO (Shanghai) Biotechnology Co., Ltd. Fetal bovine serum (FBS) and TRIzol reagent were purchased from Shanghai Life‐iLab Biotechnology Co., Ltd. and Takara Biotechnology (Dalian) Co., Ltd., respectively. The SYBR Green qPCR Kit (Fast HS) and PrimeScript RT Reagent Kit were provided by Guangzhou Mutual Success Technology Co., Ltd. Reference standards, including naringenin, chlorogenic acid, quercetin, galloyl paeoniflorin, protocatechuic acid, gallic acid, and albiflorin (purity 98%), were purchased from Keyzhun Pharmaceutical Technology (Shanghai) Co., Ltd. Chromatographic solvents, including methanol, acetonitrile, and formic acid, were of LC–MS grade and purchased from Thermo Fisher Scientific (USA).

### 2.3. Experimental Animals

A total of seven healthy male specific pathogen‐free (SPF) Sprague–Dawley (SD) rats, weighing 220–250 g, were obtained from Hunan SJA Laboratory Animal Co., Ltd. (Hunan, China; License No. SCXK (Xiang) 2024‐0009). The rats were housed in a controlled environment maintained at a temperature of 22 ± 2°C and a relative humidity of 55% ± 5% under a 12‐h light–dark cycle. All rats were provided with ad libitum access to standard laboratory chow and water and were acclimatized to the facility for 1 week prior to experimental procedures. The experimental protocols were approved by the Animal Ethics Committee of Zunyi Medical University (Ethics approval No. ZMU21‐2503‐307).

## 3. Methods

### 3.1. Preparation of RYD Extract

RYD was prepared in accordance with traditional clinical decoction procedures. Briefly, the constituent botanical raw materials were accurately weighed as follows: 24 g of *A. membranaceus*; 15 g each of *C. pilosula*, *A. macrocephala*, *R. glutinosa*, *P. tenuifolia*, *P. lactiflora*, *L. chuanxiong*, *S. ningpoensis*, and *P. lobata*; 12 g of citri reticulatae pericarpium; and 9 g each of *A. sinensis*, *P. cocos*, *S. chinensis*, *S. miltiorrhiza*, *D. nobile*, and *O. japonicus*. Subsequently, the mixed botanical materials (total weight: 210 g) were immersed in distilled water (1:10, w/v, 2100 mL) for 30 min and boiled for 2 h. The extract was filtered while hot and concentrated under reduced pressure to a final volume of 210 mL (equivalent to 1.0 g of crude drug per mL), then stored at 4°C. To prepare the final test solution, an aliquot of the concentrated extract was transferred to a centrifuge tube and centrifuged at 12,000 rpm for 15 min to remove insoluble particulates, and the resulting supernatant was harvested.

### 3.2. Study on the Material Basis of RYD Against Angina Pectoris

#### 3.2.1. Preparation of Drug‐Containing Serum

Prior to drug administration, baseline blood samples were collected from the seven rats to prepare blank serum. To prepare the medicated serum, the rats were intragastrically administered RYD extract at a dose of 10 g/kg body weight (equivalent to twice the standard adult clinical dosage). Subsequently, blood samples (∼0.5 mL per time point) were sequentially collected from the orbital venous sinus at 5 min, 15 min, 30 min, 1 h, 3 h, and 6 h post‐administration. After clotting at room temperature for 30 min, the blood samples were centrifuged at 6000 rpm for 10 min at 4°C to separate the serum, which was then stored at −80°C. Blank serum was prepared identically from identical animals prior to dosing. Before analysis, the serum samples were thawed at 4°C, and a 50 μL aliquot of serum was transferred into a 1.5 mL microcentrifuge tube, mixed with 200 μL of 50% methanol (v/v) for protein precipitation, and thoroughly vortexed for 3 min. After centrifugation at 12,000 rpm for 15 min at 4°C, the supernatant was collected as the test solution for analysis.

#### 3.2.2. Composition Analysis of RYD and Its Drug‐Containing Serum

The chemical profiles of the RYD test solution, medicated serum samples, and blank serum samples were analyzed using a UHPLC–MS/MS system to collect mass spectrometry data. Chromatographic separation was performed on a Waters ACQUITY UPLC BEH C18 column (2.1 × 100 mm, 1.7 μm) maintained at 30°C. The mobile phase consisted of 0.1% aqueous formic acid (A) and acetonitrile (B) with the gradient elution profile optimized as follows: 0–1 min, 0% B; 1–20 min, 0%–10% B; 20–30 min, 10%–15% B; 30–35 min, 15%–18% B; 35–48 min, 18%–28% B; 48–58 min, 18%–65% B; 58–60 min, 65%–100% B; 60–68.1 min, 100% B; 68.1–71 min, 0% B. The flow rate was 0.3 mL/min, and the injection volume was 2 μL.

Mass spectrometric detection was performed using an electrospray ionization (ESI) source operating simultaneously in both positive and negative ion scanning modes. The source parameters were set as follows: ion source Gas 1/Gas 2, 50 psi; curtain gas, 35 psi; and source temperature, 500°C. The ion spray voltages were 5000 V (positive) and −4500 V (negative), and the mass scan range was m/z 50–1250. Data were acquired using SCIEX OS software (Version 3.1.6.4) and analyzed with PeakView software (Version 1.2). Chemical constituents in the decoction and their absorbed prototype compounds in the serum were identified based on accurate mass, MS/MS fragments (MS1/MS2 spectra), and retention times compared with literature data and online repositories, including MassBank, MS‐DIAL, and the GNPS platform.

### 3.3. Network Pharmacology and Molecular Docking Study

#### 3.3.1. Screening of Serum Components of RYD and Key Targets for Angina Pectoris

The Canonical SMILES notations of the identified serum components were obtained from PubChem (https://pubchem.ncbi.nlm.nih.gov). Their potential therapeutic targets were predicted using SwissTargetPrediction (http://swisstargetprediction.ch/), with the organism species restricted to “*Homo sapiens.*” After removing duplicate entries, the candidate targets for the absorbed components were obtained. Concurrently, angina pectoris–related therapeutic targets were retrieved from four public repositories, including GeneCards (http://www.genecards.org/), TTD (http://bidd.nus.edu.sg/group/ttd/ttd.asp), DrugBank (https://www.drugbank.ca/), and PharmGKB (https://www.pharmgkb.org/), using “angina pectoris” as the search query. Subsequently, the gene names of targets obtained from these four databases were integrated, and duplicates were eliminated to generate a consolidated disease‐associated target dataset. The component targets and disease‐related targets were then mapped using Venny 2.1.0 (https://bioinfogp.cnb.csic.es/tools/venny/) to identify the intersection targets, which were defined as the key candidate targets of RYD against angina pectoris.

#### 3.3.2. Construction of the Protein–Protein Interaction (PPI) Network

The intersection targets were imported into the STRING database (https://string-db.org/cgi/input.pl) to construct an interactive network. The minimum required interaction score was set to a high‐confidence threshold of 0.900, the organism was selected as “*Homo sapiens,*” and disconnected nodes were excluded from the network. Subsequently, the interaction data were imported into Cytoscape software (Version 3.10.2) for network visualization and topological analysis to identify hub/core therapeutic targets based on network parameters.

#### 3.3.3. GO Function and KEGG Pathway Enrichment Analysis

The intersection targets were submitted to the Bioinformatics online platform (a comprehensive data visualization wizard, http://www.bioinformatics.com.cn/) for functional annotation. GO enrichment analysis was performed, with statistical significance defined as a *p*‐value less than 0.05. The top 10 significantly enriched terms within the molecular function (MF), biological process (BP), and cellular component (CC) categories were visualized using a histogram. For KEGG pathway enrichment, the top 10 significantly enriched pathways (with a *p*‐value less than 0.05) were selected and visualized as a bubble chart.

#### 3.3.4. Construction of the “Component–Target” Network

The active constituents of RYD and the intersection targets were imported into Cytoscape software (Version 3.10.2) to construct and visualize the compound–target (C–T) interaction network, characteristically highlighting the multicomponent and multitarget regulatory paradigm of RYD.

#### 3.3.5. Molecular Docking

To evaluate the binding affinities between key ligands and receptors, molecular docking simulations were performed using the primary active constituents and the core targets exhibiting high degree centrality values. The three‐dimensional (3D) structures of the small molecules were obtained from the TCMSP database, and the 3D crystal structures of the core targets were downloaded from the Protein Data Bank (PDB) and UniProt databases. Subsequently, receptor preparation—including the removal of water molecules and co‐crystallized heteroatoms, as well as the addition of polar hydrogen atoms—was executed using PyMOL and AutoDockTools (Version 1.5.6). Finally, molecular docking was performed using AutoDock software, and the optimal binding conformations were visualized with PyMOL.

### 3.4. In Vitro Cell Validation

#### 3.4.1. Culture of AC16 Cells

Human AC16 cells were routinely cultured in complete medium (DMEM‐F12 basic medium containing 10% FBS). Culture conditions were set at 37°C, 5% CO_2_ and saturated humidity.

#### 3.4.2. Detection of AC16 Cell Proliferation Viability by CCK‐8 Assay

The AC16 cell suspension was harvested, counted with a hemocytometer under a microscope, and seeded into 96‐well plates at a density of 2 × 10^4^ cells per well, with six replicate wells per group. Each well received 100 μL of the cell suspension, while the peripheral edge wells were filled with 100 μL of sterile PBS to minimize edge effects resulting from media evaporation. Subsequently, the plates were incubated overnight to allow cell adherence. The culture medium was then replaced with 100 μL of complete medium containing graded concentrations of naringenin (10 nmol/L, 50 nmol/L, 100 nmol/L, 200 nmol/L, 500 nmol/L, 1 μmol/L, 10 μmol/L, and 20 μmol/L). The treated cells were incubated for an additional 24 h.

Following the 24‐h incubation, the supernatant was discarded, and 100 μL of medium containing 10% (v/v) CCK‐8 reagent was added to each well. After a 1‐h incubation at 37°C in the dark, the optical density (OD) at 450 nm was measured using a microplate reader. Cell viability was calculated to determine the optimal naringenin concentrations for subsequent experiments.

#### 3.4.3. Grouping and Administration

Cells were allocated into five experimental groups: a normal control group, a model group, and three naringenin‐treated groups (low, medium, and high doses).

For the normal control group, cells were cultured exclusively in 2 mL of complete medium. To establish the oxidative stress model, cells in the remaining four groups were exposed to hydrogen peroxide (H_2_O_2_) at a final concentration of 50 μmol/L supplemented with complete medium to a total volume of 2 mL. After a 4‐h incubation, the H_2_O_2_‐containing medium was removed, and the cells were washed with PBS to terminate the reaction.

Subsequently, cells in the normal control and model groups were replenished with 2 mL of complete medium, while cells in the three treatment groups were incubated with 2 mL of complete medium supplemented with the corresponding concentrations of naringenin. All cells were harvested after an additional 24 h of incubation.

#### 3.4.4. Detection of mRNA Expression Levels of Target Genes by Real‐Time Quantitative (RT‐qPCR)

Total RNA was extracted from cells treated with naringenin and H_2_O_2_ using TRIzol reagent. RT‐qPCR was performed using synthesized cDNA as the template and SYBRRIME qPCR Kit (Fast HS) as fluorescent dye to detect the relative expression of EGFR and AKT1 genes. Primers were designed using Primer 5 software and synthesized by Sangon Biotech (Shanghai) Co., Ltd., as shown in Table 1.

**TABLE 1:** Primer sequences of core target proteins.

Target gene	Upstream primer sequence	Downstream primer sequence
EGFR	TTGCCGCAAAGTGTGTAAC	GTCACCCCTAAATGCCACCG
AKT1	AGCGACGTGGCTATTGTGAAG	GCCATCATTCTTGAGGAGGAAGT
GAPDH	CAATGACCCCTTCATTGACC	GACAAGCTTCCCGTTCTCAG

## 4. Results

### 4.1. Phytochemical Profiling and Serum Pharmacochemical Analysis

The acquired high‐resolution mass spectrometry datasets of RYD and its medicated serum were processed and annotated utilizing the GNPS and MassBank databases. Consequently, a total of 179 chemical constituents were tentatively identified or characterized from the decoction extract, comprising 30 flavonoids, 26 organic acids, 9 amino acids, 8 alkaloids, 8 glycosides, 8 monoterpenoids, 6 phenylethanoid glycosides, 5 phenylpropanoids, 4 phenolic acids, 3 oligosaccharides, and 72 other compounds. The representative total ion chromatograms (TICs) of the decoction extract are illustrated in Figure 1, and the detailed mass specification data of these 179 constituents are listed in Table S1.

**FIGURE 1:**
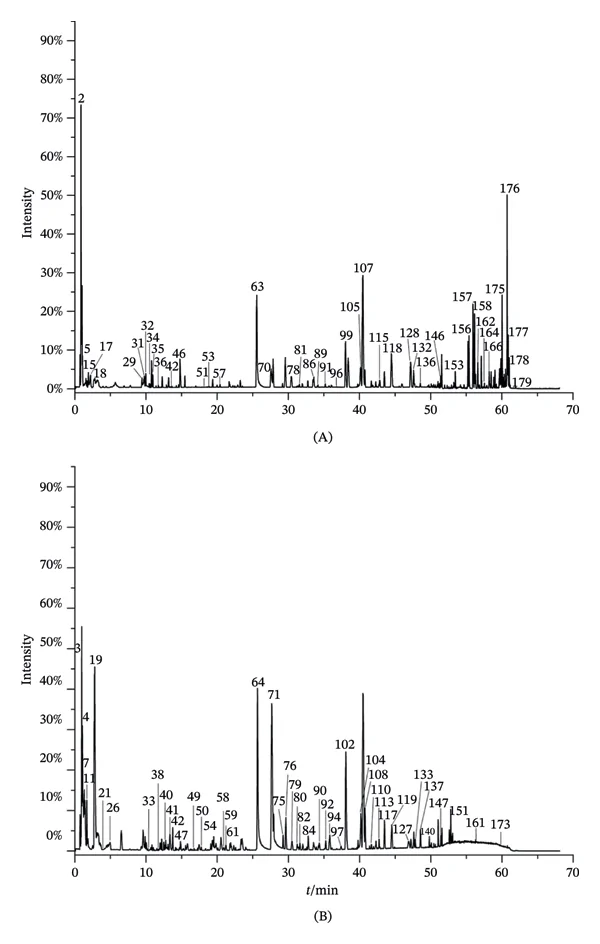
Extracted ion chromatogram of Renshen Yangying decoction extract. (A) Positive ion mode; (B) negative ion mode.

Through a comparative analysis against reference standards, blank serum, and medicated serum samples, 37 absorbed prototype constituents were successfully characterized in vivo (Figure S1). These serum‐migrating components structurally spanned various chemical classes, including flavonoids, organic acids, oligosaccharides, alkaloids, and amino acids. Tracing their botanical attributes revealed that 8 components originated from *P. cocos*, 6 from *A. macrocephala*, 5 from *S. chinensis*, 4 from *A. sinensis*, 4 from *C. pilosula*, 4 from *P. lobata*, 2 from *A. membranaceus*, 2 from *C. reticulata*, 1 from *D. nobile*, and 1 from *L. chuanxiong*. Collectively, these results fundamentally demonstrate the complex “multi‐component” material basis of RYD, providing solid chemical evidence that aligns with the TCM paradigm of multitarget synergistic therapeutic actions.

### 4.2. Network Pharmacology Analysis

#### 4.2.1. Identification of RYD Candidate Targets Against Angina Pectoris

Candidate therapeutic targets for the identified RYD serum components were predicted using the SwissTargetPrediction webserver, yielding a total of 1451 targets, which were consolidated into 614 unique targets after redundancy elimination. Concurrently, angina pectoris–associated targets were retrieved using the query “angina pectoris” across four public repositories, identifying 6545 targets from GeneCards, 30 from TTD, 38 from DrugBank, and 2 from PharmGKB. Integration of these datasets followed by duplicate removal yielded 2393 consolidated disease‐associated targets. Subsequently, the 614 component‐related targets and the 2393 disease targets were cross‐referenced using Venny 2.1.0, revealing 282 intersection targets that represent the core candidate targets of RYD in the treatment of angina pectoris (Figure S2A).

#### 4.2.2. Construction and Analysis of the PPI Network

The 282 intersection targets were imported into the STRING database to generate a PPI network (Figure S2B). Subsequently, the raw PPI network was imported into Cytoscape (Version 3.10.2) for network visualization and topological analysis. Ultimately, essential hub targets were screened based on the median topological thresholds of closeness centrality (0.0018), betweenness centrality (273.36), and degree centrality (43.18). The targets were ranked by their degree centrality values, thereby identifying *AKT1* (degree = 156), *TNF* (degree = 148), *BCL2* (degree = 125), *STAT3* (degree = 124), and *EGFR* (degree = 124) as the top hub therapeutic targets of RYD against angina pectoris (Figure 2).

**FIGURE 2:**
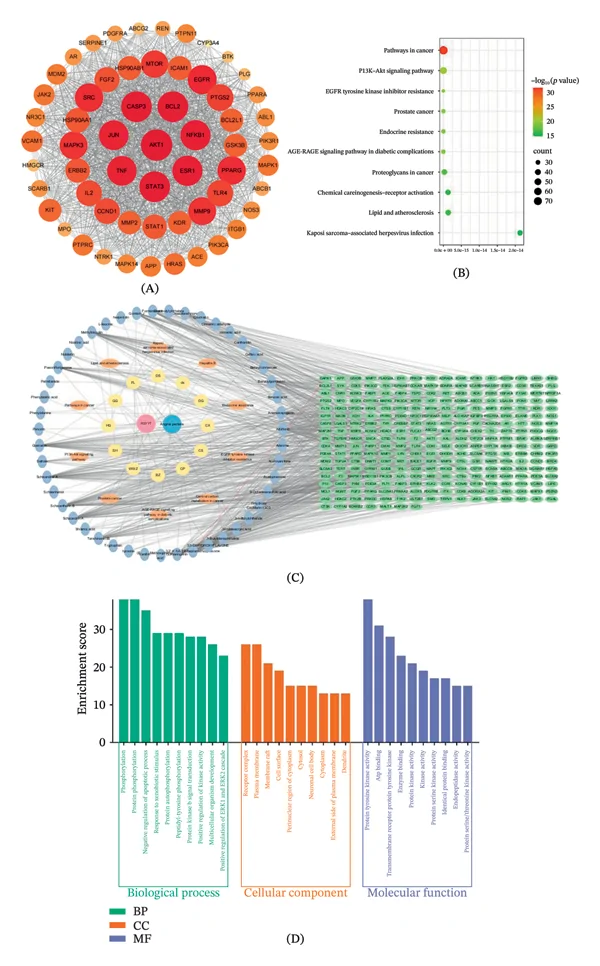
Network pharmacology analysis of Renshen Yangying decoction. (A) Optimization of the protein–protein interaction network of targets of Renshen Yangying decoction against angina pectoris; (B) GO (A) and KEGG (B) enrichment bar charts; (C) component–target network diagram; (D) network relationship diagram of component–target–enriched pathways. Note: Pink: Renshen Yangying decoction; blue: angina; yellow: HQ: Astragali Radix (*Astragalus*); WWZ: Schisandrae Chinensis Fructus; SH: Dendrobium; BZ: Atractylodis Macrocephalae Rhizoma; CX: Chuanxiong Rhizoma; DS: Salviae Miltiorrhizae Radix et Rhizoma; DG: Angelicae Sinensis Radix; CP: Citri Reticulatae Pericarpium; CS: Paeoniae Radix Rubra; DS: Codonopsis Radix; FL: Poria; GG: Puerariae Lobatae Radix; orange: access; purple: component; green: gene targets.

#### 4.2.3. GO and KEGG Enrichment Analysis

To elucidate the biological functions of the candidate targets, GO enrichment analysis was performed, identifying a total of 610 significantly enriched GO terms. These functional annotations included 205 BP terms, predominantly featuring the epidermal growth factor receptor signaling pathway, the insulin‐like growth factor receptor (IGF‐R) signaling pathway, and the platelet‐derived growth factor receptor‐beta signaling pathway. Additionally, 121 CC terms were enriched (e.g., receptor complex, plasma membrane, and cell surface), alongside 284 MF terms, such as histone H3Y41 kinase activity, histone H2AXY142 kinase activity, and protein tyrosine kinase activity (Figure 2A).

Furthermore, KEGG pathway enrichment analysis revealed that the intersection targets were significantly mapped to 127 pathways, including pathways in cancer, the PI3K–Akt signaling pathway, and EGFR tyrosine kinase inhibitor resistance (Figure 2D). Based on gene counts and statistical significance, the top 10 significantly enriched pathways were selected for visualization (Figure 2B). Collectively, these results demonstrate that RYD alleviates angina pectoris via a holistic “multi‐component, multi‐target, and multi‐pathway” synergistic paradigm.

#### 4.2.4. Network Pharmacology Analysis

To globally visualize the multitarget regulatory mechanisms, a comprehensive compound–target–pathway (C–T–P) network was constructed using Cytoscape (Version 3.10.2) based on the 37 serum components, 282 candidate targets, and the top 10 significantly enriched signaling pathways (Figure 2C). Network topology analysis highlighted schisantherin B, schisandrol A, naringenin, and quercetin as the major active constituents owing to their high node degrees. Concurrently, AKT1, TNF, BCL2, STAT3, and EGFR served as the core potential targets, while pathways in cancer, the PI3K–Akt signaling pathway, and EGFR tyrosine kinase inhibitor–related pathways emerged as the pivotal therapeutic axes through which RYD modulates angina pectoris.

#### 4.2.5. Molecular Docking of Key Constituents With Core Targets

As detailed in Section 3.3.4, active ingredients were docked against core targets, and binding energies were analyzed; lower binding energies indicate greater receptor–ligand affinity and more stable docking conformations (Figure 3). The results demonstrate that the binding affinities of AKT1 with each active ingredient are strong (i.e., < −5.9 kcal/mol), indicating that AKT1 is a key target. PyMOL software was then used to visualize the docking results of AKT1 with schisantherin B, schisandrol A, naringenin, and quercetin (Figure 4).

**FIGURE 3:**
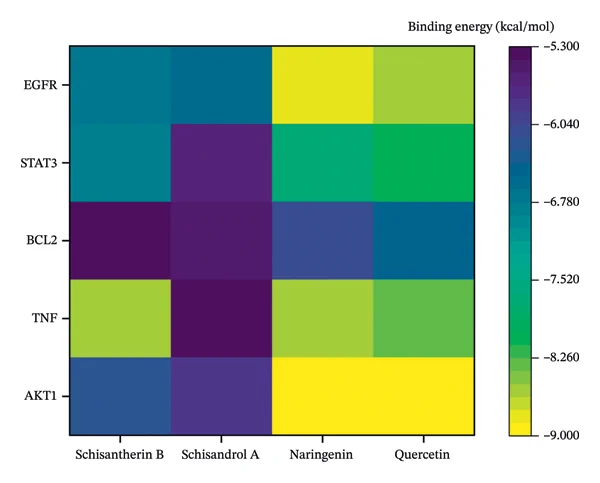
Binding energies of molecular docking between potential active components and AKT1 protein (kcal/mol).

**FIGURE 4:**
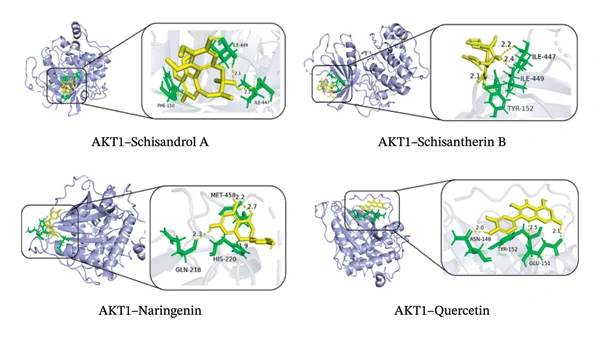
Visualization of molecular docking results between potential active components and AKT1 protein.

### 4.3. In Vitro Experimental Validation

#### 4.3.1. Cytotoxicity Assessment of Naringenin in AC16 Cells

To establish a safe dosing regimen for naringenin, a CCK‐8 assay was conducted to evaluate its cytotoxicity toward AC16 cells. The results revealed that cell viability remained completely unaffected when naringenin was administered at concentrations ranging from 50 nmol/L to 1 μmol/L (Figure S3). Consequently, the nontoxic concentrations of 100 nmol/L and 1 μmol/L were selected for subsequent anti‐inflammatory and cytoprotective experiments in this study.

#### 4.3.2. Effects of Naringenin on *EGFR* and *AKT1* mRNA Expression in Challenged AC16 Cells

Upon exposure to H_2_O_2_, the mRNA expression levels of *EGFR* and *AKT1* in the model group were markedly upregulated compared with those in the normal control group, confirming the successful establishment of the cellular injury model. Importantly, subsequent intervention with naringenin significantly and concentration‐dependently suppressed the transcription of both *EGFR* and *AKT1* (Figure 5). These results demonstrate that naringenin, a key constituent of RYD, exerts a significant attenuating effect on cellular inflammation and injury in AC16 cells.

**FIGURE 5:**
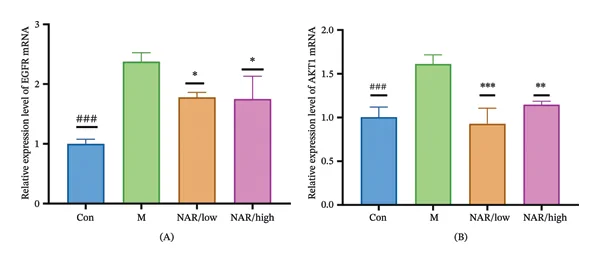
Comparison of relative mRNA expression levels of EGFR and AKT1 in AC16 cells among each group. Note: “#” indicates comparison between the model group and the normal control group; “^∗^” indicates comparison between the model group and other groups. ^##^ *p* < 0.01, ^#^ *p* < 0.05, ^∗^ *p* < 0.05, ^∗∗^ *p* < 0.01.

## 5. Discussion

In the present study, a robust UHPLC–Q‐TOF‐MS/MS–based chemical profiling strategy was deployed for the first time to comprehensively characterize the chemical constituents of RYD, resulting in the identification of 179 compounds. Building upon this baseline characterization, a subsequent analysis of medicated serum samples successfully identified 37 absorbed prototype components, including organic acids, amino acids, and alkaloids. These circulating constituents were chromatographically traced back to 10 primordial medicinal herbs, predominantly *P. cocos*, *A. macrocephala*, *S. chinensis*, and *A. sinensis*.

These findings collectively underscore that the therapeutic efficacy of RYD following oral administration is driven not by a solitary compound, but rather by a multicomponent consortium that dynamically enters the systemic circulation. Consequently, these blood‐accessible xenobiotics and their metabolites likely constitute the true bioactive material basis mediating the formula’s pharmacological effects [14, 15]. Nevertheless, because natural product chemistries undergo rapid in vivo biotransformation, tracking and identifying their full metabolite profiles remains a formidable challenge.

Network pharmacology results indicate that RYD attenuates angina pectoris primarily by modulating key pathways, including the EGFR, IGF‐R, and platelet‐derived growth factor receptor‐β (PDGFR‐β) signaling pathways. Among these cascades, the EGFR signaling pathway activates downstream AKT and ERK nodes to orchestrate essential cardioprotective effects [16, 17]. Concurrently, the PI3K–Akt signaling pathway, acting as a critical hub governing cell survival, proliferation, metabolism, and the inflammatory cascade, demonstrates distinct protective effects on the cardiovascular system by suppressing cardiomyocyte apoptosis and promoting angiogenesis [18]. Furthermore, the PDGFR‐β signaling pathway facilitates myocardial repair by promoting cardiomyocyte proliferation and vascular maturation in the infarct area, while the IGF‐R signaling pathway exerts synergistic protective effects by activating downstream survival kinases [19]. Taken together, these bioinformatic insights suggest that RYD improves disease phenotypes by harmoniously modulating core proteins such as AKT1, TNF, BCL2, STAT3, and EGFR, further confirming that its antianginal action obeys a classic “multi‐component, multi‐target, and multi‐pathway” paradigm.

Molecular docking experiments further substantiated that the potential active components of RYD (naringenin, schisantherin B, schisandrol A, and quercetin) exhibit strong binding affinity to these core targets. To validate the reliability of these in silico predictions, this study conducted in vitro experiments using human AC16 cardiomyocytes. AC16 cells, derived from primary adult ventricular tissue, possess the intact genetic background of human cardiomyocytes coupled with the capacity for indefinite proliferation; hence, they have been widely adopted to recapitulate cardiovascular disease models, including myocardial ischemia/reperfusion injury [20–22]. Given their high phenotypic fidelity to primary cardiomyocytes, they serve as a translationally relevant model for evaluating the clinical potential of RYD. Our CCK‐8 results indicated that at naringenin concentrations of 100 nmol/L and 1 μmol/L, cell viability was not significantly affected. Crucially, in the H_2_O_2_‐challenged cellular model, naringenin intervention remarkably downregulated the transcriptional levels of both EGFR and AKT1 mRNA compared with the untreated model group. This protective effect directly corroborates the computational predictions from our network pharmacology and molecular docking analyses, identifying naringenin as a pivotal cardioprotective driver within the formula.

By integrating advanced untargeted metabolomics with network pharmacology, this study successfully narrowed down the vast chemical space of RYD to a clear landscape of core therapeutic targets. Our docking assays revealed intriguing multiligand, multireceptor dynamics, where schisantherin B exhibited good binding affinity to TNF, schisandrol A to EGFR, naringenin to AKT1, and quercetin to both AKT1 and EGFR. This structural complementarity highlights that distinct chemical scaffolds within RYD are specifically tailored to hit divergent protein nodes in vivo. Interestingly, our in vitro data showed that naringenin significantly suppressed AKT1 and EGFR transcription at both 100 nmol/L and 1 μmol/L without showing strict dose‐dependency, suggesting a potential saturation kinetics or a ceiling effect common in multitarget natural polyphenols. Nevertheless, due to the lack of molecular dynamics (MD) simulations and animal functional rescue experiments, the precise in vivo spatial‐temporal mechanisms cannot be fully clarified. Therefore, future studies should integrate gene knockout models and multiomics technologies to thoroughly analyze the dynamic regulatory network of these signaling pathways, thereby providing more robust evidence‐based support for the clinical translation of RYD.

## 6. Conclusions

This study provides the first comprehensive characterization of the chemical profile and absorbed material basis of RYD via UHPLC–Q‐TOF‐MS technology, successfully identifying 179 chemical constituents in the extract and 37 absorbed prototype components in medicated serum. Chemical classification revealed that both the intact decoction and its circulating counterparts predominantly consist of flavonoids, lignans, and organic acids. Through an integrated network pharmacology and molecular docking approach, naringenin, schisantherin B, schisandrol A, and quercetin were identified as the primary bioactive constituents driving the antianginal efficacy of RYD. These pivotal xenobiotics target a regulatory network of core hub proteins (including AKT1, TNF, BCL2, and EGFR), which are deeply involved in the PI3K–Akt cascade and EGFR tyrosine kinase inhibitor resistance pathways. Crucially, in vitro functional assays demonstrated that naringenin, a key representative constituent, significantly attenuated cellular injury and inflammation in H_2_O_2_‐challenged AC16 cardiomyocytes, effectively corroborating our bioinformatic and structural docking predictions. Collectively, these findings establish a robust, network‐based mechanistic framework for RYD, offering insights into its holistic “multi‐component, multi‐target” paradigm and supporting the evidence‐based modernization of TCM‐derived antianginal therapeutics.

## Author Contributions

Ya Huang, conceptualization, methodology, software, data curation, validation, investigation, visualization. Tao Xiong, writing–original draft, validation, formal analysis, investigation, visualization. Haixia Qiu, resource, investigation, data curation. Rui Cheng, investigation, data curation. Yule Luo, resources. Xingdong Wu, resources, supervision, writing–review and editing, project administration.

## Funding

This study was supported by the Administration of Traditional Chinese Medicine of Guizhou Province, QZYY‐2024–197.

## Ethics Statement

The experiments received ethics approval from the Experimental Animal Ethics Committee of Zunyi Medical University (No. ZMU21‐2503‐307).

## Conflicts of Interest

The authors declare no conflicts of interest.

## Supporting Information

Additional supporting information can be found online in the Supporting Information section.

## Supporting information


**Supporting Information** Appendix A. Supporting data. Supporting data for this article can be found online.

## Data Availability

The data that support the findings of this study are available on request from the corresponding author. The data are not publicly available due to privacy or ethical restrictions.
