# Supplementary material for: Study on the Active Constituents and Underlying Mechanisms of Renshen Yangying Decoction Against Angina Pectoris Based on Integrated Serum Pharmacochemistry, Network Pharmacology, Molecular Docking, and Experimental Validation
Source: J Anal Methods Chem. 2026 Nov 3;2026:6325730. doi: 10.1155/jamc/6325730 (PMC13728912; doi:10.1155/jamc/6325730)
Supplement: Supplementary file 1 [file JAMC-2026-6325730-s001.docx]

**Study on the Active Constituents and Underlying Mechanisms of Renshen Yangying Decoction against Angina Pectoris Based on Integrated Serum Pharmacochemistry, Network Pharmacology, Molecular Docking and Experimental Validation**

**Ya Huang^a，b1^，Tao Xiong^b1^，Haixia Qiu ^b^，Rui Cheng^b^，Yule Luo^b^，Xingdong Wu^a，b*^**

*a Jinsha County Chinese Medicine Hospital, Jinsha, Guizhou 551800, China*

*b School of Pharmacy, Zunyi Medical University, Zunyi, Guizhou, 563000, China*

* Corresponding author: Tel./fax: +86 28609676

E-mail address: wuxingdong@zmu.edu.cn (Xingdong Wu).

1 These authors made equal contributions to this work.

**Figure S1 The extracted ion chromatogram of serum containing the drug**

Note: A is blank serum, B is drug-containing serum. 1 is positive ion mode, 2 is negative ion mode.

A

B

**Figure S2 Venn diagram of the intersection between component targets of Renshen Yangying Decoction and disease targets (A) and the PPI network structure diagram of potential targets (B)**

 **Figure S3 Relationship between drug concentration and cell survival rate**

**Table S1 Identification results of the components in Renshen Yangying Decoction Extracts and Serum**

| No. | tR(min) | Name | Molecular formula | Ion mode | Measured m/z | Theoretical m/z | Mass error (ppm) | | Fragment ions m/z |
| --- | --- | --- | --- | --- | --- | --- | --- | --- | --- |
| 1 | 0.822 | Arginine* | C6H14N4O2 | [M+H]^+^ | 175.1191 | 175.1191 | 0.8 | 130.0979,116.0712,70.0645 | |
| 2 | 0.863 | Choline* | C5H13NO | [M+H]^+^ | 104.1074 | 104.10699 | 3.9 | 104.1069,60.0804,58.0646 | |
| 3 | 0.929 | Gluconic acid* | C6H12O7 | [M-H]^-^ | 195.0506 | 195.05103 | 3.4 | 195.0504,177.0419,159.0294,141.0181,75.0085,59.0137 | |
| 4 | 1.031 | Raffinose | C18H32O16 | [M-H]^-^ | 503.1628 | 503.16176 | 2.1 | 503.1649,383.1205,221.0674,179.0564,89.0246 | |
| 5 | 1.039 | (E)-deca-2-ene-4,-diyne-1,10-diol-1-O-β-D apiofuranosyl-(1→6)-β-D-glu-copyranoside | C21H29O11 | [M+H]^+^ | 458.1774 | 458.17826 | -1.9 | 116.0709,70.0653 | |
| 6 | 1.046 | Proline* | C5H9NO2 | [M+H]^+^ | 116.0705 | 116.07061 | -0.9 | 70.0652,68.0515 | |
| 7 | 1.10 | Sucrose* | C12H22O11 | [M-H]^-^ | 341.1097 | 341.10894 | 2.2 | 341.1093,179.0563,161.0461,149.0456,119.0351,89.0244,59.0137 | |
| 8 | 1.112 | Quinic acid* | C7H12O6 | [M-H]^-^ | 191.0566 | 191.05611 | 2.6 | 191.0564,127.0407,111.0455,87.0091,57.0346 | |
| 9 | 1.165 | D-Ribose | C5H10O5 | [M-H]^-^ | 149.0460 | 149.04555 | 3.0 | 149.0460,89.0252,71.0136,59.0130 | |
| 10 | 1.249 | Glycine betaine* | C5H11NO2 | [M+H]^+^ | 118.0867 | 118.08626 | 3.8 | 118.0890,72.0808,55.0543 | |
| 11 | 1.601 | Shikimic acid | C7H10O5 | [M-H]^-^ | 173.0463 | 173.04555 | 4.4 | 137.0251,93.0491,65.0395 | |
| 12 | 1.702 | Homoproline* | C6H11NO2 | [M+H]^+^ | 130.0867 | 130.08626 | 3.4 | 130.0875,84.0805,69.0579,56.0491 | |
| 13 | 1.704 | Mycosporine or its isomers | C11H19NO6 | [M+H]^+^ | 262.1296 | 262.12851 | 4.1 | 262.1308,244.1209,216.1245,198.1159,161.0706,84.0813,72.0816 | |
| 14 | 1.799 | Adenine | C5H5N5 | [M+H]^+^ | 136.0617 | 136.06177 | -0.5 | 136.0622,119.0356,92.0247,65.0135,55.0292 | |
| 15 | 1.990 | Nicotinic acid | C6H5NO2 | [M+H]^+^ | 124.0399 | 124.0393 | 4.8 | 124.0395,96.0456,80.0498,53.0387 | |
| 16 | 2.052 | Benzaldehyde | C7H6O | [M+H]^+^ | 107.0496 | 107.04914 | 4.3 | 107.0494,91.0540,79.0576,77.0386 | |
| 17 | 2.116 | Cytidine* | C9H13N3O5 | [M+H]^+^ | 244.0926 | 244.0928 | -0.8 | 244.1133,226.1071,182.0958,112.0509 | |
| 18 | 2.65 | L-Pyroglutamic acid* | C5H7NO3 | [M+H]^+^ | 130.0501 | 130.04987 | 1.8 | 130.0515,84.0442,56.0493 | |
| 19 | 2.771 | Citric acid* | C6H8O7 | [M-H]^-^ | 191.0202 | 191.01973 | 2.5 | 191.0202,111.0089,87.0088,85.0296 | |
| 20 | 3.352 | L-Leucine or its isomers* | C6H13NO2 | [M+H]^+^ | 132.1023 | 132.10191 | 3 | 86.0967,69.0710 | |
| 21 | 4.120 | Debenzoylpaeoniflorin or its isomers | C16H24O10 | [M-H]^-^ | 375.1315 | 375.12967 | 4.9 | 375.1310,345.1198,195.0662,165.0569 | |
| 22 | 4.159 | Aucubin | C15H22O9 | [M-H]^-^ | 345.1204 | 345.11911 | 3.7 | 299.2017,225.0049,165.0568,147.0448,123.0459 | |
| 23 | 4.280 | Tyrosine* | C9H11NO3 | [M+H]^+^ | 182.0817 | 182.08117 | 2.9 | 136.0769,107.0502,91.0544 | |
| 24 | 4.296 | Benzoic acid* | C7H6O2 | [M+H]^+^ | 123.0445 | 123.04406 | 3.6 | 81.0344,77.0389 | |
| 25 | 4.67 | Uracil | C4H4N2O2 | [M+H]^+^ | 113.035 | 113.03455 | 3.9 | 113.0359,96.0087,96.0313,70.0290 | |
| 26 | 4.928 | Uridine* | C9H12N2O6 | [M-H]^-^ | 243.0628 | 243.06226 | 2.2 | 243.0665,152.0359,122.0243,110.0252,82.0304,66.0349 | |
| 27 | 5.209 | 9-Amino-1,3,9-nonanetricarboxylic acid | C12H21NO6 | [M+H]^+^ | 276.1459 | 276.14416 | 6.3 | 276.1488,230.1414,132.1035 | |
| 28 | 6.446 | Gallic acid # | C7H6O5 | [M+H]^+^ | 171.0296 | 171.0288 | 4.7 | 153.0190,135.0098,107.0140,81.0342 | |
| 29 | 9.382 | Adenosine | C10H13N5O4 | [M+H]^+^ | 268.1044 | 268.10403 | 1.4 | 136.0618,119.0358 | |
| 30 | 9.73 | 2-Aminostyrene* | C8H9N | [M+H]^+^ | 120.0806 | 120.08078 | -1.5 | 120.0816,103.0547,91.0546,77.0388,65.0398 | |
| 31 | 9.760 | Phenylalanine* | C9H11NO2 | [M+H]^+^ | 166.0870 | 166.08626 | 4.5 | 149.0584,120.0810,103.054,77.0386 | |
| 32 | 9.967 | Guanosine | C10H13N5O5 | [M+H]^+^ | 284.0996 | 284.09895 | 2.3 | 152.0571,135.0310,110.0361 | |
| 33 | 10.376 | 1-Galloyl-glucose | C13H16O10 | [M-H]^-^ | 331.0684 | 331.06707 | 4.0 | 331.0711,211.0523,168.9880,124.0166 | |
| 34 | 10.564 | Safrole | C10H10O2 | [M+H]^+^ | 163.0755 | 163.07536 | 0.9 | 130.0649,117.0699,105.0702,91.0562,80.0492 | |
| 35 | 10.836 | 2-methyl-1H-indol-7-yl-β-D-mannopyranoside or its isomers | C15H19NO6 | [M+H]^+^ | 310.1290 | 310.12851 | 1.6 | 310.1259,292.1224,264.1253,166.0869,132.0824,120.0814 | |
| 36 | 11.736 | codonopsinol B | C13H19NO4 | [M+H]^+^ | 254.1398 | 254.13868 | 4.4 | 161.0617,146.0369,121.0656,86.0605,76.0604 | |
| 37 | 11.796 | Cinnamaldehyde | C9H8O | [M+H]^+^ | 133.0653 | 133.06479 | 3.8 | 115.0545,105.0711,77.0392 | |
| 38 | 11.84 | D-Pantothenic acid* | C9H17NO5 | [M-H]^-^ | 218.1042 | 218.1034 | 3.7 | 146.0830,88.0408,71.0502 | |
| 39 | 11.853 | Protocatechuic acid # | C7H6O4 | [M-H]^-^ | 153.0196 | 153.01933 | 1.7 | 153.0204,109.0298,108.0217,91.0186,65.0033 | |
| 40 | 12.760 | Rehmannioside D | C27H42O20 | [M-H]^-^ | 685.2228 | 685.21967 | 4.6 | 685.2258,341.1089,179.0588,89.0249 | |
| 41 | 13.338 | Sorbitol hexaacetate | C18H26O12 | [M-H]^-^ | 433.1357 | 433.13515 | 1.3 | 433.1208,205.0721,143.0726,129.0559 | |
| 42 | 13.63 | Acetovanillone | C9H10O3 | [M+H]^+^ | 167.0711 | 167.07027 | 5.0 | 167.0711,149.0601,121.0652,91.0542,77.0392 | |
| 43 | 13.741 | Dihydrocatalpol | C15H24O10 | [M-H]^-^ | 363.1314 | 363.12967 | 4.8 | 363.1361,201.0777,183.0680,165.0569,157.0509,59.0138 | |
| 44 | 14.314 | Gardoside | C16H22O10 | [M-H]^-^ | 373.1153 | 373.11402 | 3.4 | 373.1153,211.0666,193.0529,149.0611,123.0454,59.0142 | |
| 45 | 14.669 | Succinoadenosine* | C14H17N5O8 | [M-H]^-^ | 382.1020 | 382.10044 | 4.1 | 382.1030,266.0884,206.0692,134.0475 | |
| 46 | 14.790 | Indole-3-acrylic acid* | C11H9NO2 | [M+H]^+^ | 188.0709 | 188.07061 | 1.6 | 188.0723,146.0611,144.0823,118.0656,91.0548 | |
| 47 | 14.843 | Tryptophan* | C11H12N2O2 | [M-H]^-^ | 203.0832 | 203.0826 | 2.9 | 203.0832,142.0672,116.0513,74.0248 | |
| 48 | 15.176 | Sibiricose A6 | C23H32O15 | [M+H]^+^ | 549.1815 | 549.1814 | 0.2 | 549.1865,369.1211,177.0610 | |
| 49 | 16.733 | Decaffeoylverbascoside | C20H30O12 | [M-H]^-^ | 461.1675 | 461.16645 | 2.3 | 461.1671,315.1091,297.1013,161.0458,135.0456 | |
| 50 | 17.777 | 6'-O-Galloyldebenzoylpaeoniflorin or its isomers | C23H28O14 | [M-H]^-^ | 527.1421 | 527.14063 | 2.8 | 497.1323,313.0592,271.0471,169.0152 | |
| 51 | 18.019 | (+)-Catechin hydrate | C15H14O6 | [M+H]^+^ | 291.0859 | 291.08631 | -1.4 | 291.1152,207.0696,139.0402,123.0460 | |
| 52 | 18.635 | (1R,4S,6R)-1,3,3-Trimethyl-2-oxabicyclo[2.2.2]oct-6-yl 6-O-β-D-glucopyranosyl-β-D-glucopyranoside | C22H38O12 | [M-H]^-^ | 493.2309 | 493.22905 | 3.7 | 493.2347,447.2272,285.1731,179.0578,161.0435,119.0320,59.0142 | |
| 53 | 18.878 | N-isopentenyl-6-hydrox ydendroxinium | C22H34NO4 | [M+H]^+^ | 377.2575 | 377.25606 | 3.8 | 377.2600,261.1643,193.0341,171.1522,100.0758 | |
| 54 | 19.488 | Puerarin-6-C-glucoside | C27H30O14 | [M-H]^-^ | 577.1579 | 577.15628 | 2.8 | 577.1577,487.1260,457.1156,429.1197,415.1205,295.0622,266.0593,237.0559, 223.0408, | |
| 55 | 20.06 | 10-griselinosidic acid | C17H22O12 | [M-H]^-^ | 417.1052 | 417.10385 | 3.2 | 417.1061,241.0738,152.0121,108.0220 | |
| 56 | 20.302 | Chlorogenic acid # | C16H18O9 | [M+H]^+^ | 355.1032 | 355.10236 | 2.4 | 163.0398,145.0296,135.0451,117.0340,89.0389 | |
| 57 | 20.355 | 7-Hydroxymethylcoumarin | C9H6O3 | [M+H]^+^ | 163.0395 | 163.03897 | 3.2 | 145.0305,135.0442,117.0338,89.0389,77.0393,63.0233 | |
| 58 | 20.883 | Oxypaeoniflorin or its isomers | C23H28O12 | [M-H]^-^ | 495.1522 | 495.1508 | 2.8 | 495.1523,,333.1020,165.0560,137.0246 | |
| 59 | 21.22 | Caffeic acid | C9H8O4 | [M-H]^-^ | 179.0356 | 179.03498 | 3.4 | 179.0428,135.0455,107.0496,89.0413 | |
| 60 | 22.117 | 5-Hydroxyferulic acid | C15H24O5 | [M-H]^-^ | 283.1563 | 283.1551 | 4.2 | 283.1554,208.9596,139.1140,81.0339 | |
| 61 | 22.491 | Isoacteoside | C21H28O13 | [M-H]^-^ | 487.1479 | 487.14571 | 4.5 | 487.1469,279.0902,251.0555,179.0367,161.0285,135.0475 | |
| 62 | 25.174 | Neorehmannioside E | C21H34O10 | [M-H]^-^ | 445.2100 | 445.20792 | 4.7 | 445.2092,265.1437,235.1350,89.0245 | |
| 63 | 25.602 | Cantharidin | C10H12O4 | [M+H]^+^ | 197.0818 | 197.08084 | 4.9 | 179.0688,161.0616,133.0645,123.0817,79.0551 | |
| 64 | 25.611 | Puerarin* | C21H20O9 | [M-H]^-^ | 415.1048 | 415.10346 | 3.2 | 415.1054,325.0736,295.0621,267.0669,2253.0519 | |
| 65 | 25.636 | Formononetin | C16H12O4 | [M-H]^-^ | 267.0670 | 267.06628 | 2.7 | 267.0711,252.0433,91.0193 | |
| 66 | 26.124 | Riboflavin* | C17H20N4O6 | [M+H]^+^ | 377.1471 | 377.14556 | 4.1 | 243.0906,198.0679,172.0889 | |
| 67 | 26.529 | Sibiricoses A5 | C22H30O14 | [M-H]^-^ | 517.1576 | 517.15628 | 2.6 | 517.1576,193.0504,160.0192 | |
| 68 | 26.799 | Gardenoside | C17H24O11 | [M-H]^-^ | 403.1264 | 403.12459 | 4.5 | 403.1300,223.0608,179.0711,139.0398,119.0509,101.0248 | |
| 69 | 26.802 | Swertisin | C22H22O10 | [M+H]^+^ | 447.1302 | 447.12857 | 3.6 | 447.1340,429.1218,411.1127,327.0911,297.0789, | |
| 70 | 27.500 | Mirificin-4′-O-glucoside isomers | C32H38O18 | [M+H]^+^ | 711.2131 | 711.21309 | 0 | 711.2187,579.1786,399.1131,351.0858 | |
| 71 | 27.514 | Rutin-2,4′-dimethyl ether | C28H32O16 | [M-H]^-^ | 623.1647 | 623.16176 | 4.7 | 623.1696,533.1290,503.1288,413.0877,383.0772 | |
| 72 | 27.536 | trans-4-Methoxycinnamic acid | C10H10O3 | [M+H]^+^ | 179.071 | 179.07027 | 4.1 | 179.0702,161.0617,151.0748,133.0663,118.0428,105.0704,77.0389 | |
| 73 | 27.562 | 4-Ethoxybenzaldehyde | C9H10O2 | [M+H]^+^ | 151.0759 | 151.07536 | 3.6 | 123.0845,105.0705,77.0392,67.0551 | |
| 74 | 27.906 | Puerarin 6''-O-xyloside | C26H28O13 | [M-H]^-^ | 547.1480 | 547.14571 | 4.2 | 547.1479,415.1073,397.0951,325.0743,295.0625,267.0675,253.0528,149.0615 | |
| 75 | 29.192 | Prunin | C21H22O10 | [M-H]^-^ | 433.1161 | 433.11402 | 4.8 | 433.1080,271.0632,151.0057 | |
| 76 | 29.578 | 6-Methoxykaempferol 3-O-rhamnoside | C22H22O11 | [M-H]^-^ | 461.1103 | 461.10894 | 3.0 | 415.1050,325.0719,253.0513,132.0220 | |
| 77 | 29.995 | Pterosupin isomers | C21H24O10 | [M-H]^-^ | 435.1317 | 435.12967 | 4.7 | 273.0795,137.0260,109.0298 | |
| 78 | 30.316 | Hymecromone | C10H8O3 | [M+H]^+^ | 177.0553 | 177.05462 | 3.8 | 149.0606,145.0297,134.0372,89.0388,77.0384 | |
| 79 | 30.465 | Echinacoside | C35H46O20 | [M-H]^-^ | 785.2540 | 785.25097 | 3.9 | 785.2552,623.2226,161.0251,133.0307 | |
| 80 | 31.173 | Sibiricaxanthone A | C24H26O14 | [M-H]^-^ | 537.1270 | 537.12498 | 3.8 | 537.1263,387.0754,267.0310,243.0313 | |
| 81 | 31.513 | Vitexin | C21H20O10 | [M+H]^+^ | 433.1153 | 433.11292 | 5.5 | 433.1177,397.0975,313.0732,284.0687,283.0635 | |
| 82 | 31.576 | 3'-Hydroxypuerarin | C21H20O10 | [M-H]^-^ | 431.0997 | 431.09837 | 3.1 | 431.1005,341.0682,311.0573,269.0464,149.0254 | |
| 83 | 32.165 | Senkyunolide K | C12H16O3 | [M+H]^+^ | 209.1180 | 209.11722 | 3.7 | 209.1194,191.1086,163.1119,149.0907,135.1188 | |
| 84 | 32.749 | Vitexin-O-xyloside | C26H28O14 | [M-H]^-^ | 563.1423 | 563.14063 | 3.0 | 563.1434,341.0688,311.0570,283.0627 | |
| 85 | 33.009 | Irisxanthone | C20H20O11 | [M+H]^+^ | 437.1094 | 437.10784 | 3.6 | 401.0918,317.0678,287.0596,259.0625 | |
| 86 | 33.582 | Polygalaxanthone III | C25H28O15 | [M+H]^+^ | 569.1501 | 569.1501 | 0.0 | 569.1514,437.1132,419.1027,341.0693,317.0693,287.0586,271.0636 | |
| 87 | 33.760 | 1,2,3,6-Tetragalloylglucose | C34H28O22 | [M-H]^-^ | 787.1031 | 787.09995 | 2.0 | 787.1050,635.0938,617.0828,465.0737 | |
| 88 | 33.908 | Eriocitrin | C27H32O15 | [M-H]^-^ | 595.1688 | 595.16684 | 3.3 | 595.1694,287.0569,151.0044,135.0452,107.0147 | |
| 89 | 34.079 | Limonin | C26H30O8 | [M+H]^+^ | 471.2013 | 471.20134 | -0.1 | 471.2042,425.1984,213.0919,161.0596,95.0146 | |
| 90 | 34.260 | Sibirioside A | C21H28O12 | [M-H]^-^ | 471.1529 | 471.1508 | 4.5 | 471.1555,323.1027,189.0520,161.0614,147.0461,103.0557 | |
| 91 | 35.166 | Quercetin*# | C15H10O7 | [M+H]^+^ | 303.0512 | 303.04993 | 4.2 | 303.0528,229.0505,201.0549,153.0202 | |
| 92 | 35.188 | Rutin | C27H30O16 | [M-H]^-^ | 609.1477 | 609.14611 | 2.6 | 609.1488,301.0364,300.0292 | |
| 93 | 35.541 | 3',6'-Digalloylpaeoniflorin | C37H36O19 | [M-H]^-^ | 783.1813 | 783.1778 | 4.5 | 783.1810,465.0701,313.0561,169.0156 | |
| 94 | 35.792 | Galloylpaeoniflorin # | C30H32O15 | [M-H]^-^ | 631.1688 | 631.16684 | 3.1 | 631.1692,509.1340,491.1229,479.1222,463.1284,271.0474 | |
| 95 | 35.900 | Tenuifoliside B | C30H36O17 | [M-H]^-^ | 667.1904 | 667.18797 | 3.6 | 461.1341,235.0566,205.0522,190.0281,137.0257 | |
| 96 | 36.728 | Perlolyrine | C16H12N2O2 | [M+H]^+^ | 265.0983 | 265.09715 | 4.3 | 265.1077,247.0897,219.0936,206.0856,205.0775,167.0534 | |
| 97 | 37.347 | Isoacteoside | C29H36O15 | [M-H]^-^ | 623.1999 | 623.19814 | 2.8 | 623.2007,461.1711,161.0250,135.0456 | |
| 98 | 37.782 | Rehmapicroside | C16H26O8 | [M-H]^-^ | 345.1571 | 345.15549 | 4.7 | 345.1580,165.0932,119.0354 | |
| 99 | 38.005 | Naringenin* # | C15H12O5 | [M+H]^+^ | 273.0770 | 273.07575 | 4.5 | 273.0773,153.0196,147.0448,119.0482,91.0553 | |
| 100 | 38.010 | Narirutin | C27H32O14 | [M+H]^+^ | 581.1885 | 581.18648 | 3.5 | 273.0769,195.0305,153.0194,85.02863,71.0493 | |
| 101 | 38.212 | 3-Butylidenephthalide | C12H14O3 | [M+H]^+^ | 207.1020 | 207.10157 | 2.2 | 207.1024,189.0929,171.0796,147.0444,133.0664,128.0527,105.0332,91.0548 | |
| 102 | 38.468 | Jionoside B1 | C37H50O20 | [M-H]^-^ | 813.2851 | 813.28227 | 3.5 | 813.2884,637.2426,491.1811,175.0411,160.0178 | |
| 103 | 39.849 | Diosmin | C28H32O15 | [M+H]^+^ | 609.1825 | 609.1814 | 1.8 | 609.1885,463.1282,301.0732 | |
| 104 | 40.101 | Albiflorin # | C23H28O11 | [M-H]^-^ | 479.1576 | 479.15589 | 3.6 | 479.1584,283.0757,121.0300,77.0396 | |
| 105 | 40.140 | Paeoniflorigenone | C17H18O6 | [M+H]^+^ | 319.1192 | 319.11762 | 5 | 319.1066,105.0703,77.0392 | |
| 106 | 40.433 | Hesperetin | C16H14O6 | [M+H]^+^ | 303.0879 | 303.08631 | 5.2 | 177.0565,153.0193,149.0601,145.0296,117.0355,89.0398 | |
| 107 | 40.463 | Hesperidin | C28H34O15 | [M+H]^+^ | 611.1996 | 611.19705 | 4.1 | 303.0874,195.0304,177.0555,153.0190 | |
| 108 | 40.731 | Neorehmannioside D | C21H34O9 | [M-H]^-^ | 429.2151 | 429.21301 | 4.9 | 429.2163,267.1634,249.1524,187.1494 | |
| 109 | 40.746 | 3,6'-Disinapoly sucrose | C34H42O19 | [M-H]^-^ | 753.2270 | 753.22475 | 3.0 | 753.2278,547.1693,529.1590,367.1041,223.0624,205.0512,190.0281 | |
| 110 | 41.616 | Rosmarinic acid | C18H16O8 | [M-H]^-^ | 359.0784 | 359.07724 | 3.2 | 197.0475,179.0360,161.0255,135.0456,133.0302,72.9930 | |
| 111 | 42.238 | Limocitrin-3-O-HMG-β-glucoside | C29H32O17 | [M+H]^+^ | 653.1728 | 653.17123 | 2.4 | 653.3422,347.0791,332.0562,127.0399 | |
| 112 | 42.524 | Pueroside C | C24H26O10 | [M+H]^+^ | 475.1614 | 475.15987 | 3.2 | 313.1112,295.0979,267.1036,107.0495 | |
| 113 | 42.705 | Angoroside C | C36H48O19 | [M-H]^-^ | 783.2744 | 783.2717 | 3.4 | 783.2756,607.2288,461.1680,193.0514,175.0507,160.0174 | |
| 114 | 42.760 | 8-p-Coumaroylharpagide | C24H30O12 | [M-H]^-^ | 509.1671 | 509.16645 | 5.0 | 509.1709,463.2199,307.0846,163.0397,145.0291 | |
| 115 | 42.769 | Ononin | C22H22O9 | [M+H]^+^ | 431.1353 | 431.13366 | 3.8 | 431.1397,269.0826,251.0713,237.0554,118.0418 | |
| 116 | 43.063 | 6-O-E-Feruloylajugol | C25H32O12 | [M-H]^-^ | 523.1843 | 523.1821 | 4.2 | 523.1880,193.0508,175.0417,134.0386 | |
| 117 | 43.471 | Tenuifoliside A | C31H38O17 | [M-H]^-^ | 681.2060 | 681.20362 | 3.5 | 443.1220,281.0692,263.0574,239.0571,179.0360,137.0249 | |
| 118 | 44.40 | Denobilone B | C15H16O4 | [M+H]^+^ | 261.1131 | 261.11214 | 3.7 | 261.1102,175.9936,137.0607,122.0364,91.0552,65.0393 | |
| 119 | 44.505 | 3,5-Dihydroxyflavone | C15H10O4 | [M-H]^-^ | 253.0513 | 253.05063 | 2.6 | 253.0516,225.0560,160.0148,135.0100,117.0349,93.0334 | |
| 120 | 44.53 | Tenuifoliose M | C65H82O37 | [M-H]^-^ | 1453.4462 | 1453.44622 | 0.7 | 1161.3567,1119.3463,933.6246,841.5363 | |
| 121 | 44.530 | Tenuifoliose G | C66H84O38 | [M-H]^-^ | 1483.4581 | 1483.45678 | 1.5 | 1161.3563,1119.3449,1101.3152,753.2434 | |
| 122 | 44.539 | （6αR,11αR）-3-Yydroxy-9,10- Dimethoxy-pterocarpan* | C17H16O5 | [M+H]^+^ | 301.1082 | 301.10705 | 3.8 | 301.1099,167.0716,147.0445 | |
| 123 | 44.610 | L-bornyl 7-O-α-arabinofuranosyl (1→6)-β-D-glucopyranoside | C21H36O10 | [M-H]^-^ | 447.2247 | 447.22357 | 2.5 | 447.2331,315.1702,161.0458,71.0147 | |
| 124 | 44.835 | Nomilinic acid glucoside | C34H48O16 | [M-H]^-^ | 711.2890 | 711.28696 | 2.9 | 711.2910,651.2697,607.2790 | |
| 125 | 45.882 | Rehmannioside | C31H40O15 | [M-H]^-^ | 651.2324 | 651.22944 | 4.5 | 651.2299,505.1745,287.0281,193.0508,175.0403,161.0229 | |
| 126 | 46.15 | Salvianolic acid A | C26H22O10 | [M-H]^-^ | 493.1163 | 493.11402 | 4.6 | 295.0665,185.0289,135.0459,72.9929 | |
| 127 | 46.854 | Salvianolic acid B | C36H30O16 | [M-H]^-^ | 717.1479 | 717.14611 | 2.5 | 717.1501,519.0950,339.0524,321.0416,295.0626,277.0512,185.0255 | |
| 128 | 47.101 | Denbinobin | C16H12O5 | [M+H]^+^ | 285.0771 | 285.07575 | 4.7 | 285.0784,270.0544,253.0522,213.0567,137.0249,91.0583 | |
| 129 | 47.132 | Tenuifoliside C | C35H44O19 | [M-H]^-^ | 767.2416 | 767.2404 | 4.2 | 767.2453,529.1608,367.1042,265.0752,237.0740,223.0622,205.0503,190.0453 | |
| 130 | 47.385 | Coumarin | C9H6O2 | [M+H]^+^ | 147.0447 | 147.04406 | 4.4 | 91.0544,65.0393,55.9354 | |
| 131 | 47.444 | Tenuifoliose L | C67H84O38 | [M-H]^-^ | 1495.4591 | 1495.45678 | 2.3 | 1203.3676,1185.3586,1161.3570,1143.3472,1081.3385,795.2406 | |
| 132 | 47.593 | Poncirin | C28H34O14 | [M+H]^+^ | 595.2055 | 595.20213 | 5.7 | 287.0933,263.0576,195.0295,153.0194,85.0291 | |
| 133 | 47.814 | Salvianolic acid C | C26H20O10 | [M-H]^-^ | 491.1006 | 491.09837 | 4.5 | 491.0993,293.0469,265.0523,179.0359,159.0469,135.0459 | |
| 134 | 47.909 | 3-Butylidenephthalide | C12H12O2 | [M+H]^+^ | 189.0917 | 189.09101 | 3.7 | 171.0836,119.0869,91.0537,72.0818,55.9360 | |
| 135 | 48.452 | Tenuifoliose T | C56H70O32 | [M-H]^-^ | 1253.3785 | 1253.37774 | 1.5 | 1131.3503,1091.3299,985.3214,823.2485,319.0871 | |
| 136 | 48.489 | Cinnamic Acid | C9H8O2 | [M+H]^+^ | 149.0604 | 149.05971 | 4.7 | 103.0558,77.0392,65.0390,51.0227 | |
| 137 | 48.542 | Isoharpagoside | C24H30O11 | [M-H]^-^ | 493.1739 | 493.17154 | 4.8 | 345.1202,295.0731,239.0693,183.0654,165.0574,147.0460,103.0562 | |
| 138 | 48.573 | Tenuifoliose C | C58H72O33 | [M-H]^-^ | 1295.3891 | 1295.38831 | 1.5 | 1119.3474,1077.3308,997.3127,851.2909,145.0293 | |
| 139 | 49.627 | Neorehmannioside C | C28H40O10 | [M-H]^-^ | 535.2572 | 535.25487 | 4.4 | 535.2585,163.0417,145.0300 | |
| 140 | 49.738 | Tenuifoliose I | C59H72O33 | [M-H]^-^ | 1307.3876 | 1307.38831 | 0.3 | 1161.3600,1119.3448,1101.3279,1039.3097,325.0892 | |
| 141 | 50.009 | Tenuifoliose B | C60H74O34 | [M-H]^-^ | 1337.3992 | 1337.39887 | 1.1 | 1161.3575,1119.3468,1101.34643,1077.3564,1039.3147,175.0417 | |
| 142 | 50.252 | Tenuifoliose O | C61H76O35 | [M-H]^-^ | 1367.4107 | 1367.40944 | 1.7 | 1245.3508,1191.3698,1149.3669,1027.3153,175.0401 | |
| 143 | 50.608 | Polygalasaponin ⅩⅩⅧ | C53H84O24 | [M-H]^-^ | 1103.5277 | 1103.52798 | 0.8 | 1103.5289,455.3177,425.3085,337.1160 | |
| 144 | 50.645 | Tetradecylcitric acid | C20H36O7 | [M+H]^+^ | 389.2563 | 389.25338 | 7.5 | 389.2584,371.2412,330.2098,327.2074,311.2154,284.2029,244.1725,232.1715,113.0710 | |
| 145 | 50.855 | Onjisaponin Tf | C59H94O28 | [M-H]^-^ | 1249.5876 | 1249.58589 | 2.2 | 1249.5929,1025.5371,455.3205,425.3050,154.8747 | |
| 146 | 51.404 | Benzoylpaeoniflorin | C30H32O12 | [M+H]^+^ | 585.1988 | 585.19665 | 3.7 | 607.1860,485.1477,341.1017,219.0643 | |
| 147 | 51.434 | Tenuifoliose A | C62H76O35 | [M-H]^-^ | 1379.4082 | 1379.40944 | -0.1 | 1203.3672,1161.3568,1143.3482,1039.3111,145.0294 | |
| 148 | 51.584 | Tenuifoliose N | C63H78O36 | [M-H]^-^ | 1409.4227 | 1409.42 | 2.7 | 1233.3762,1191.3443,1173.3667,825.2455,179.0580 | |
| 149 | 51.741 | 9,12,13-Trihydroxy-10,15-octadecadienoic acid | C18H32O5 | [M-H]^-^ | 327.2188 | 327.2177 | 3.4 | 327.2185,229.1456,211.1344,183.1387,171.1039 | |
| 150 | 52.681 | α-Hydroxylinolenic acid | C18H30O3 | [M+H]^+^ | 295.2280 | 295.22677 | 4.2 | 277.2195,224.1236,165.1282,149.1372,69.0736 | |
| 151 | 52.777 | Pinellic acid* | C18H34O5 | [M-H]^-^ | 329.2336 | 329.23335 | 4.1 | 329.2333,229.1453,211.1337,183.1392,171.1028,139.1124,127.1124,99.0812 | |
| 152 | 52.911 | Isosinensetin | C20H20O7 | [M+H]^+^ | 373.1296 | 373.12818 | 3.8 | 357.1000,343.0848,327.0525,315.0905,181.0153,153.0198 | |
| 153 | 53.497 | Atractylenolide III | C15H20O3 | [M+H]^+^ | 249.1495 | 249.14852 | 3.9 | 249.1518,231.1412,213.1305,175.0785,91.0564 | |
| 154 | 54.288 | Cryptochlorogenic acid | C30H46O3 | [M+H]^+^ | 455.3528 | 455.35197 | 1.8 | 455.3563,437.3415,297.2247,161.1375 | |
| 155 | 54.925 | 23-Hydroxybetulinic acid | C30H48O4 | [M+H]^+^ | 473.3646 | 473.36254 | 4.4 | 473.3668,437.3450,297.2193,143.1071 | |
| 156 | 55.340 | Nobiletin | C21H22O8 | [M+H]^+^ | 403.1401 | 403.13874 | 3.4 | 403.1416,388.1197,373.0949,327.0894,211.0264 | |
| 157 | 55.920 | Schisandrin* | C24H32O7 | [M+H]^+^ | 433.2240 | 433.22208 | 4.4 | 415.2149,384.1976,369.1736,346.1451 | |
| 158 | 56.153 | 3-Methoxynobiletin* | C22H24O9 | [M+H]^+^ | 433.1510 | 433.14931 | 3.9 | 433.1531,418.1296,417.1219,403.1057 | |
| 159 | 56.255 | Phenylacetic acid | C8H8O2 | [M+H]^+^ | 137.0601 | 137.05971 | 3 | 137.0613,119.0516,93.0697,77.0392 | |
| 160 | 56.337 | Atractylenolide I | C15H18O2 | [M+H]^+^ | 231.1393 | 231.13796 | 5.8 | 231.1396,185.1339,161.0592,157.1308,149.0622,143.0846,129.0755,105.0700,91.0554 | |
| 161 | 56.337 | (9Z)-9-Octadecenedioic acid* | C18H32O4 | [M-H]^-^ | 311.2232 | 311.22278 | 3.6 | 311.2232,293.2168,275.2017,223.1708,87.0470 | |
| 162 | 56.663 | Gomisin D | C28H34O10 | [M+H]^+^ | 531.2229 | 531.22247 | 0.8 | 531.2269,401.1662,383.1529,341.1040,337.1138,85.0561 | |
| 163 | 56.811 | Gomisin J | C22H28O6 | [M+H]^+^ | 389.1964 | 389.19587 | 1.4 | 389.1983,325.1492,287.0945,273.0759,137.0595,95.0871 | |
| 164 | 57.593 | 3-n-Butylphthalide | C12H14O2 | [M+H]^+^ | 191.1073 | 191.10666 | 3.4 | 191.1088,173.0984,128.0630,91.0549 | |
| 165 | 57.778 | Schisantherin B* | C28H34O9 | [M+H]^+^ | 515.229 | 515.22756 | 2.8 | 515.2369,385.1691,370.1476,355.1602,343.1209,323.1317,316.0967,267.0685 | |
| 166 | 58.273 | γ-Schisandrin | C23H28O6 | [M+H]^+^ | 401.1966 | 401.19587 | 1.8 | 401.2010,386.1771,370.1802,316.0967 | |
| 167 | 58.489 | Benzoylgomisin H | C30H34O8 | [M+H]^+^ | 523.2338 | 523.23264 | 2.2 | 523.2356,386.1683,315.1271 | |
| 168 | 59.043 | Hydroxyatractylon | C15H20O2 | [M+H]^+^ | 233.1536 | 233.15361 | 0 | 233.1590,215.1433,187.1513,177.0930,159.0813,91.0545 | |
| 169 | 59.059 | schinsanlignone A | C24H30O7 | [M+H]^+^ | 431.2076 | 431.20643 | 2.7 | 400.1830,372.1641,356.1665 | |
| 170 | 59.346 | 7-[4-(11-hydroxy-undecyloxy)-phenyl]-7-pyridin-3-yl hept-6-enoic acid ethyl ester* | C31H45NO4 | [M+H]^+^ | 496.3432 | 496.34214 | 2.1 | 496.3454,478.3338,184.0750,104.1079 | |
| 171 | 59.634 | Schisanhenol | C23H30O6 | [M+H]^+^ | 403.2118 | 403.21152 | 0.7 | 403.2119,388.1927,340.1640,302.1131,287.0975,243.0703,185.0829 | |
| 172 | 59.675 | Schisantherin A* | C30H32O9 | [M+H]^+^ | 537.2127 | 537.21191 | 1.5 | 537.2145,437.1612,325.1204,299.0954,227.2019 | |
| 173 | 59.973 | 9(R),10(S)-Epoxy-12(Z)-octadecenoic acid* | C18H32O3 | [M-H]^-^ | 295.2265 | 295.22787 | 4.5 | 295.2290,195.1387,171.1033,96.9642 | |
| 174 | 60.002 | Cryptotanshinone | C19H20O3 | [M+H]^+^ | 297.1496 | 297.14852 | 3.6 | 297.1511,282.1285,254.0967,249.0925,193.1039,165.0701 | |
| 175 | 60.065 | Atractylenolide IV* | C17H24O3 | [M+H]^+^ | 277.1805 | 277.17982 | 2.4 | 277.1826,221.1188,193.1244,165.0560,137.0609,105.0713 | |
| 176 | 60.755 | Phthalic acid* | C16H22O4 | [M+H]^+^ | 279.1604 | 279.15909 | 4.7 | 149.0241,121.0289,93.0330,65.0379 | |
| 177 | 60.907 | Schisandrin A* | C24H32O6 | [M+H]^+^ | 417.2277 | 417.22717 | 1.3 | 417.2314,402.2054,386.2103,371.1889,347.1524,316.1338,301.1102 | |
| 178 | 61.022 | Tanshinone IIA | C19H18O3 | [M+H]^+^ | 295.1342 | 295.13287 | 4.5 | 295.1345,280.1111,277.1243,262.1004,252.0788,249.1297,234.1047,221.1332 | |
| 179 | 61.910 | Hexadecanamide* | C16H33NO | [M+H]^+^ | 256.2644 | 256.26349 | 3.5 | 256.2657,102.0917,88.0768,57.0700 | |

Note: * indicates components that enter the bloodstream; # indicates components verified by standard reference substanc
